# Tenotomy

**Published:** 1841-07

**Authors:** 


### TENOTOMY.

We had the pleasure of witnessing this operation a short time since by Dr. Thompson, of Hartsville, Tenn., assisted by Drs. Debow and Duvall of the same place. The subject was a little boy, 10 years of age, the son of Mr. Dalton, in whom an abscess in the ham, some years before, had produced a considerable shortening of the gastrocnemii muscles, so that the lad walked on his toes. The tendo-Achillis was divided, and the foot brought at once nearly to its natural position. The most perfect success promised to attend the operation. We would call the attention of our professional brethren to the distortions of the extremities demanding this operation. They are to be met with in almost every neighborhood, and their remedy by tenotomy constitutes one of the most striking and pleasing triumphs of modern Surgery.

